# Transformation of Ammonium Azide at High Pressure and Temperature

**DOI:** 10.3390/ma13184102

**Published:** 2020-09-15

**Authors:** Guozhao Zhang, Haiwa Zhang, Sandra Ninet, Hongyang Zhu, Keevin Beneut, Cailong Liu, Mohamed Mezouar, Chunxiao Gao, Frédéric Datchi

**Affiliations:** 1 State Key Laboratory of Superhard Materials, Jilin University, Changchun 130012, China; z2012gz@163.com; 2 Institut de Minéralogie, de Physique des Matériaux et de Cosmochimie (IMPMC), Sorbonne Université, CNRS UMR 7590, MNHN, 4 Place Jussieu, F-75005 Paris, France; haiwa.zhang@outlook.com (H.Z.); sandra.ninet@sorbonne-universite.fr (S.N.); keevin.beneut@sorbonne-universite.fr (K.B.); 3 School of Physics and Electronic Engineering, Linyi University, Linyi 276005, China; hongyangzhu@jlu.edu.cn; 4 Shandong Key Laboratory of Optical Communication Science and Technology, School of Physical Science and Information Technology of Liaocheng University, Liaocheng 252059, China; cailong_liu@jlu.edu.cn; 5 European Synchrotron Radiation Facility, BP 220, F-38043 Grenoble CEDEX, France; mezouar@esrf.fr

**Keywords:** high energy-density materials, high pressure and temperature, Raman spectroscopy, X-ray diffraction, ammonium azide, polynitrogen compounds

## Abstract

The compression of ammonium azide (AA) has been considered to be a promising route for producing high energy-density polynitrogen compounds. So far though, there is no experimental evidence that pure AA can be transformed into polynitrogen materials under high pressure at room temperature. We report here on high pressure (*P*) and temperature (*T*) experiments on AA embedded in N${}_{2}$ and on pure AA in the range 0–30 GPa, 300–700 K. The decomposition of AA into N${}_{2}$ and NH${}_{3}$ was observed in liquid N${}_{2}$ around 15 GPa–700 K. For pressures above 20 GPa, our results show that AA in N${}_{2}$ transforms into a new crystalline compound and solid ammonia when heated above 620 K. This compound is stable at room temperature and on decompression down to at least 7.0 GPa. Pure AA also transforms into a new compound at similar *P*–*T* conditions, but the product is different. The newly observed phases are studied by Raman spectroscopy and X-ray diffraction and compared to nitrogen and hydronitrogen compounds that have been predicted in the literature. While there is no exact match with any of them, similar vibrational features are found between the product that was obtained in AA + N${}_{2}$ with a polymeric compound of N${}_{9}$H formula.

## 1. Introduction

At ambient conditions, nitrogen forms one of the strongest triple bonds found in nature in the diatomic molecule N${}_{2}$. When submitted to high pressures (P>110 GPa) and temperatures (T>2000 K) the N${}_{2}$ molecular crystal transforms into the extended, single-bonded cubic gauche phase of nitrogen (cg-N) [1,2]. cg-N is a high energy density material (HEDM), with a much larger energy density than conventional explosives, due to the large energy difference between single and triple bonds. Such a HEDM would be an ideal source of clean energy to be used, for example, as propellant. Unfortunately, cg-N is not recoverable at ambient conditions, since it reverts to molecular N${}_{2}$ below 42 GPa at 300 K.

Various groups have considered adding elements that could enhance the stability of nitrogen oligomers to increase the stability of polynitrogen materials at ambient conditions. Hydronitrogen (N${}_{x}$H${}_{y}$) compounds have been given particular attention, when considering that their high mass ratio of nitrogen makes them attractive for HEDM and that hydrogen could effectively passivate polymeric structures at ambient conditions [3,4,5]. Various poly-hydronitrogen compounds have been indeed predicted under high pressure, not yet observed at ambient conditions [3,4,6,7]. Depending on the composition, these structures contain infinite one-dimensional (1D)-polymeric chains (N${}_{2}$H, N${}_{3}$H, N${}_{4}$H), infinite 2D-polymeric chains (N${}_{9}$H${}_{4}$), or pentazole (N${}_{5}$H) molecules. Experiments [3,6,8,9,10] on N${}_{2}$-H${}_{2}$ mixtures observed that when compressed over 50 GPa, the two molecules react and form N-H bonds suggesting the presence of N${}_{x}$H${}_{y}$ oligomers. However, their identification has been made difficult by the disordered nature of the product(s), which results in broad Raman and IR peaks and diffuse X-ray diffraction.

Ammonium azide (AA) has been considered as a promising precursor to poly-hydronitrogen. Indeed, the double bond of the azide ion N${}_{3}^{-}$ is weaker than the triple bond of N${}_{2}$ and, thus, easier to break. Computer simulations have predicted the polymerization of nitrogen in various forms in AA compressed to pressures in excess of 60 GPa [11,12,13]. So far, experimental works have investigated AA compressed to 85 GPa at room temperature and did not report any evidence of polymerization [12,14]. Synthesizing polynitrogen compounds from the sole compression of AA would thus require extreme pressures in the 100 GPa range and is, therefore, not a practical route. Recently, a theoretical work has predicted that crystals of NH${}_{4}$N${}_{5}$ and N${}_{5}$H, both featuring all-nitrogen cyclic pentazole ions (N${}_{5}^{-}$) could be synthesized at high pressure [15]. In particular, compressing NH${}_{4}$N${}_{3}$ with N${}_{2}$ above 12.5 GPa was proposed as a route for obtaining NH${}_{4}$N${}_{5}$. In this respect, it is worth mentioning the recent synthesis of the pentazolate salts CsN${}_{5}$ [16] and LiN${}_{5}$ [17] obtained by the reaction of the respective metal or metal azide with nitrogen at high pressures and temperatures. When compared with metal pentazolate salts, NH${}_{4}$N${}_{5}$ and N${}_{5}$H would present a higher mass ratio of nitrogen, an environmentally friendly clean HEDM, and lower *P*–*T* conditions of synthesis, making it more practical for large production and more likely to recover the product at ambient conditions.

Below, we report the results of our investigations on the AA + N${}_{2}$ and pure AA systems at pressures up to 30 GPa and temperatures up to 700 K, while using Raman spectroscopy and X-ray diffraction. A transformation of the AA sample is found at 620 K for pressures above 20 GPa in both cases, but the recovered product is different. The Raman spectrum and X-ray diffraction pattern of the new phases have been measured up to 25 GPa at room temperature. We find no evidence for the predicted NH${}_{4}$N${}_{5}$ nor HN${}_{5}$ compounds. Comparison is made with other N${}_{x}$H${}_{y}$ compounds predicted stable at high *P*; while there is no exact match with any of them, similar vibrational features are found between the product obtained in AA + N${}_{2}$ with a polymeric compound of N${}_{9}$H formula.

## 2. Materials and Methods

### 2.1. Experimental Methods

AA powder samples were synthesized by a metathetical reaction of NaN${}_{3}$ and NH${}_{4}$NO${}_{3}$, as reported in previous studies [18]. A membrane diamond anvil cell (MDAC) was used to generate high pressures [19], equipped with diamond anvils of culet size of 300 μm. Re gaskets of initial thickness of 200 μm were preindented to a thickness of 35 μm, and a hole of diameter of 95 μm was laser-drilled at the center of the indent in order to serve as the sample chamber. A piece of AA sample of lateral size 40 to 50 μm was loaded in the gasket hole and embedded in N${}_{2}$ by cryogenic loading of N${}_{2}$, in the same fashion as our previous study using liquid argon [14]. The Raman spectrum of the AA sample after loading was compared to those that were measured in our previous study [14] and confirmed the absence of impurities, as shown in Figure S1 of the Supporting Information file (SI).

In Raman experiments, pressure was determined using the ruby and SrB${}_{4}$O${}_{7}$:Sm${}^{2+}$ fluorescent sensors, according to calibrations from Refs. [20,21], respectively. In XRD experiments, pressure was determined either from the measured volume of a small piece of gold powder or of solid nitrogen, while using the equations of state of Refs. [22,23], respectively.

We adopted an external heating method to heat the sample, using a cylindrical resistive heater (Watlow France, Asnières-sur-seine, France) whose internal diameter and length fit the outer dimensions of the cell, ensuring homogeneous heating. The heater is connected to a power unit and it is temperature controlled using a feedback loop. A K-type thermocouple is placed in contact with the cell close to the sample chamber in order to determine the sample temperature. The accuracy of temperature measurement is about 5 K.

Raman scattering was excited by the 514.5 nm line of an argon laser, collected by a confocal in-house optical set-up and dispersed by a HR460 (Horiba Jobin Yvon, Longjumeau, France) spectrometer of focal length 460 mm onto a water-cooled CCD camera (Andor Technology Ltd, Belfast, U. K.). The diameter of the laser spot on the sample, focused by 20X Mitutoyo objective, was about 2 μm. The Raman spectra were recorded in the range of 70 to 4800 cm${}^{-1}$ using a 1200 lines/mm grating.

XRD experiments were done in the angular dispersive mode using monochromatic X-rays at beamline ID27 of the European Radiation Synchrotron Facility (ESRF, Grenoble, France). The X-ray beam of wavelength 0.3738 Å was focussed to a spot size of 2.5 × 3 μm FWHM. Diffracted X-rays were collected by a bidimensional SX-165 CCD detector (marXperts, Norderstedt, Germany). The sample distance and position of the X-ray beam were calibrated while using a CeO${}_{2}$ powder standard. The analysis and refinement of XRD patterns were performed using the Fullprof software suite [24].

### 2.2. Theoretical Methods

Structural optimization and vibrational calculations were performed using, respectively, the first-principle plane-wave pseudo-potential density functional theory (DFT) and density functional perturbation theory (DFPT) [25,26], as implemented in the CASTEP code [27]. The ultrasoft pseudopotentials were used with exchange and correlation effects described by the generalized-gradient-approximation (GGA) [28] of Perdew–Burke–Ernzerhof. van der Waals corrections were implemented using the Tkatchenko–Scheffler method [29], and the value of the two scheme parameters *sR* and *d* were set at 0.94 and 20.0, respectively. The kinetic energy cutoff and Monkhorst-Pack k-point meshes (5 × 5 × 2 meshes) for structural optimization were set at 898 eV and 0.07 A${}^{-1}$, respectively. The self-consistent energy convergence criterium was set to less than 5.0 × 10${}^{-6}$ eV/atom, and the maximal force, stress and displacement set to be 0.01 eV/Å, 0.02 GPa and 5.0 × 10${}^{-4}$ Å, respectively. The Reflex powder diffraction module in the material studio software was used in order to calculate the XRD patterns.

### 2.3. High P-T Transformation of AA in N${}_{2}$

#### 2.3.1. Raman Experiments

We first investigated AA embedded in N${}_{2}$ compressed up to 30 GPa at room temperature using Raman spectroscopy. We observed no sign of phase transition in the Raman spectrum, the latter being consistent with previous measurements on pure AA and AA embedded in argon [12,14]. We then prepared three samples at pressures of about 15 GPa, 20 GPa, and 30 GPa, and studied them as a function of temperature in the range 300–700 K. The followed *P*–*T* paths are illustrated in Figure S2 of the SI.

At *P*∼15 GPa, the Raman spectrum was collected at 100 K steps and showed no phase transition to 600 K (see Figure S3 of the SI). At 700 K, nitrogen became liquid and the AA sample decomposed quickly and dissolved into liquid nitrogen. Only the Raman band of the N${}_{2}$ molecule stretching could be clearly observed at this temperature. Upon cooling back to 300 K, N-H stretch modes in the range 3150–3450 cm${}^{-1}$ were also detected at several positions, which are compatible with the Raman spectrum of ammonia (see Figure S4 of SI). The reasons why NH${}_{3}$ was not detected at high temperature likely comes from its dilution in the N${}_{2}$ liquid, the small AA sample volume, which was loaded as compared to that of N${}_{2}$ and weaker Raman intensity at high *T*. At 13 GPa–300 K, N${}_{2}$ and NH${}_{3}$ were solid and phase separated (hence, the inhomogeneous aspect of the sample shown in panel (a4) of Figure S3), and a stronger Raman signal from the NH${}_{3}$ solid was recorded in sample regions, where NH${}_{3}$ crystallized. Although not tested, we consider it unlikely that H${}_{2}$ was formed in the decomposition of AA, since (1) none were formed at higher pressures (see below) and (2) NH${}_{3}$ is more stable than N${}_{2}$ + H${}_{2}$ at this pressure [4,30,31]. Thus, we conclude that AA is not stable and decompose into N${}_{2}$ and NH${}_{3}$ in the presence of liquid N${}_{2}$ at 15 GPa. For this reason, we took care not to cross the N${}_{2}$ melting line in the two subsequent heating runs at 20 GPa and 30 GPa. The heating rate was in these cases 50 K/step. Since the experimental results for these two pressures are identical (see Figures S5–S7 of the SI), we only report below the results obtained on heating at 30 GPa.

**Figure 1:**
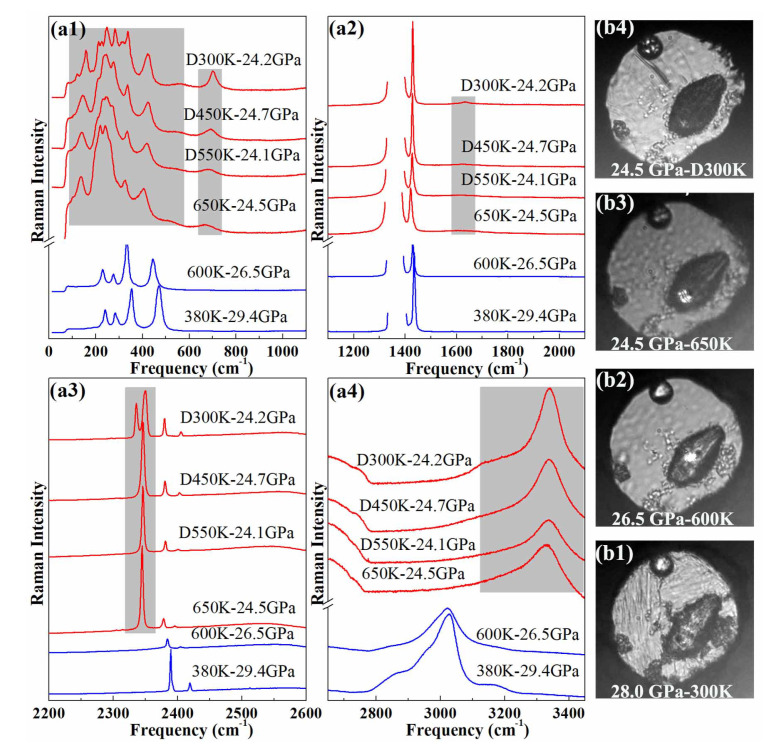
(**a**) Raman spectrum of AA + N${}_{2}$ up to 25 GPa at different temperatures. The gray colored bands emphasize the frequency windows where major changes are observed in the sample Raman spectrum. “D” indicates that the measurement was performed on decreasing temperature (**b**) Photographs of the sample chamber at different pressures and temperatures.

Upon heating the sample initally at 30 GPa at 300 K, the pressure slowly reduced to 26.5 GPa at 600 K. At 600 K–26.5 GPa, the Raman spectrum of the AA + N${}_{2}$ exhibits no significant change from the one at 380 K–29.4 GPa, apart from the expected *P*-*T* induced peak shifts and slight peak broadening, as can be seen from Figure 1. When the temperature was raised to 650 K, the Raman spectrum of the sample suddenly changed, especially in the frequency ranges colored in gray in Figure 1a. The sample light absorption also increased at this temperature [see the photographs in Figure 1b]. After holding for 10 min., some Raman peaks disappeared or weakened. After half an hour, the Raman spectrum of the sample no longer varied; it also qualitatively stayed the same when the sample was cooled down to room temperature at the same pressure.

A comparison of the sample Raman spectra before and after the high pressure heat treatment reveals major changes both in the lattice and internal modes. In particular, the N-H stretch bands discontinuously shifted by ∼300 cm${}^{-1}$ and new bands appeared in the frequency regions of N≡N stretch (2340 cm${}^{-1}$) and N=N bend (∼700 cm${}^{-1}$). The Raman spectra taken at different positions in the sample chamber (see Figure S8 of the SI) show that the reaction product is homogeneous, and the sharp molecular and lattice Raman bands strongly indicate that it is crystalline. We also note that a large amount of nitrogen remained around the sample after the transition. No evidence for the formation of H${}_{2}$ was found in the Raman spectra (see Figure S9 of the SI). As seen below, the reaction product is actually most likely composed of two phases, ammonia solid and an unknown phase referred to as phase A in the following.

The Raman spectrum of the reacted sample was collected upon decompression at 300 K (see Figure S9 of the SI). All of the modes observed after the high *P*-*T* treatment are conserved down to 7.1 GPa. Below this pressure, no measurement could be made, as lowering the force in the DAC resulted in a rapid jump to ambient pressure. When opened to air, the products synthesized in the sample chamber and nitrogen quickly volatilized. Before its complete decomposition, we could only detect the Raman spectrum characteristic of pure AA. This shows either that the high *P*–*T* reaction is reversible at a pressure between ambient and 7.1 GPa at room temperature, or that a small remnant of AA was still present and remained after all other products volatilized. This amount must be small as AA was not detected either by Raman or XRD after the reaction. AA itself eventually decomposed into volatile components and disappeared from the sample chamber after a few minutes.

**Figure 2:**
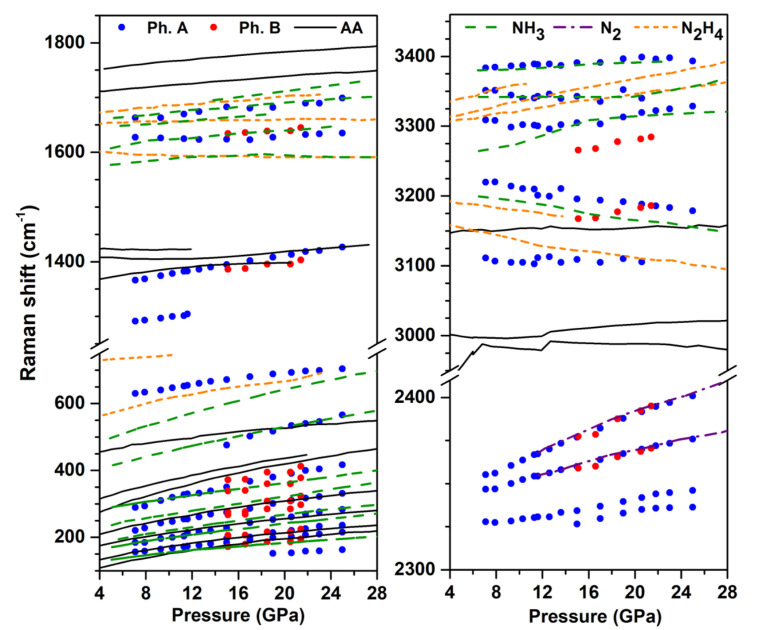
Frequency evolution with pressure of the Raman peaks of the products of the AA transformation at high *P*-*T*. The measurements were made on decompression at 300 K. The experimental data for phases A and B are represented by blue and red solid dots, respectively. The different colored lines represent literature data for the Raman modes of pure AA (black solid lines) [14], NH${}_{3}$ [33,34] (green dashed lines), N${}_{2}$ [32] (purple dashed lines), and hydrazine [35] (N${}_{2}$H${}_{4}$, orange dashed lines) solids.

Figure 2 shows the evolution of the Raman mode frequencies with pressure at 300 K, and compares them to those of the pure AA [14], N${}_{2}$ [32], NH${}_{3}$ [33,34] and hydrazine (N${}_{2}$H${}_{4}$) [35] solids obtained from literature. We recall that NH${}_{3}$ (and possibly heavier, unidentified azanes) and N${}_{2}$H${}_{4}$ (below 10 GPa on decompression) were identified as reaction products of N${}_{2}$ + H${}_{2}$ mixtures at high pressure [3,6,8,9], which justifies the present comparison. The higher frequency modes correspond to N-H stretching, and the deconvolution of the observed band reveals at least five peaks in the 3100–3400 cm${}^{-1}$ range. It can be seen that four of these N-H peaks nearly coincide with those of the pure ammonia solid reported in Refs. [33,34]; the small frequecy differences are likely due to the different sample temperature, which was 50 K in Ref. [34]. There is also a good match between the observed modes in the 1620–1700 cm${}^{-1}$ region with the reported torsion modes of NH${}_{3}$, and the same is true for the lattice mode around 500 cm${}^{-1}$ at 20 GPa, which corresponds to the A^e^ mode of NH${}_{3}$ [34]. The frequency region below 400 cm${}^{-1}$ in phase A is composed of many peaks and is more difficult to analyze in details. These observations strongly suggest that ammonia is one of the reaction products and separated in solid form from the other products. The disappearance of the N-H stretch and bending modes of NH${}_{4}^{+}$ also show that these ions disappeared and probably converted to NH${}_{3}$.

The evolution with pressure of the mode at 1420 cm${}^{-1}$ at 20 GPa is very similar to that of the N=N stretch in AA, indicating that phase A contains species with N=N double bonds. We note that in AA, the N=N stretch mode is in Fermi resonance with the bending mode of NH${}_{4}^{+}$, which is no longer the case in phase A, supporting again that NH${}_{4}^{+}$ have disappeared in this phase. Four Raman bands are observed in the N≡N stretch frequency range; two of them perfectly match those of pure solid N${}_{2}$, and the remaining two, which are located at lower frequencies (by about 20 to 40 cm${}^{-1}$), more likely belong to phase A. Finally, the band at ca. 700 cm${}^{-1}$ at 26 GPa cannot be assigned to N${}_{2}$ or NH${}_{3}$ solids and, thus, also belongs to phase A.

#### 2.3.2. XRD Experiments

**Figure 3:**
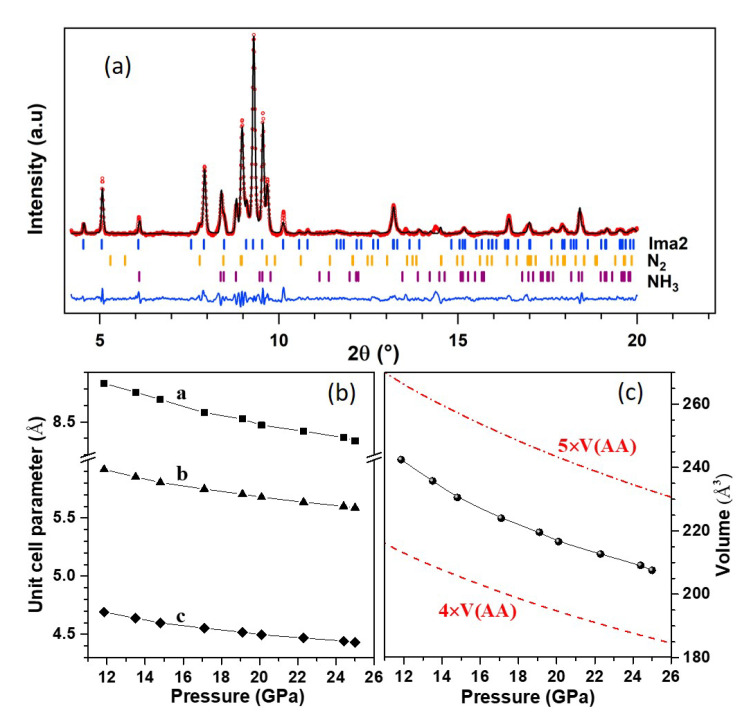
XRD pattern of the AA + N${}_{2}$ sample at 20.1 GPa–300 K after the high *P*-*T* transformation. Panel (**a**) shows the three-phase Le Bail fit of the pattern using the *Ima2* unit cell for phase A, ϵ-N${}_{2}$ (*R-3c*) and NH${}_{3}$-V (*P2${}_{1}$2${}_{1}$2${}_{1}$*). The background has been subtracted for easier viualization. The red circles are experimental data, the black line is the Le Bail fit and the blue line is the fit residual. Ticks indicate the position of the Bragg peaks for each phase. The bottom panels show the unit cell parameters (**b**) and volume (**c**) of the *Ima2* cell. The latter may be compared to the dashed and dash-dotted lines, representing, respectively, four and five times the volume of AA-II (*P2/c*).

We performed XRD measurements on the same sample as above from 25 GPa to 12 GPa at 300 K on decompression in order to explore the structure of phase A. Thanks to the small X-ray beam, XRD patterns could be collected at different positions in the sample chamber, either on the reacted sample or at a location which only contained pure solid nitrogen. Because the AA sample is surrounded by N${}_{2}$, diffraction peaks from solid N${}_{2}$ are also expected in the XRD pattern of the reacted sample. The latter at 20.1 GPa–300 K is shown in Figure 3a. Diffraction peaks of solid N${}_{2}$ (ϵ-N${}_{2}$ above 17 GPa and $\delta^{*}$-N${}_{2}$ below) and ammonia (phases V above 12 GPa and IV below) could be identified in the pattern of the reacted sample (see Figure S11 of the SI). The cell parameters and volume of N${}_{2}$ and NH${}_{3}$ obtained from the Le Bail refinement of these patterns are compared to literature [32,36] in Figure S12 of the SI, showing very good agreement in both cases. This confirms that pure solid ammonia is produced in the reaction.

Searches for the unit cell of phase A were performed with the list of non-indexed peaks in the XRD pattern at 20.1 GPa while using the DICVOL06 software [37]. This returned two possible solutions: first, an orthorhombic cell with most probable space group *Ima2*, cell parameters a=8.475(9) Å, b=5.679(4) Å, c=4.490(5) Å and volume V=216.1(4) Å${}^{3}$; second, a monoclinic cell of most probable space group *P2/m*, cell parameters a=4.738(3) Å, b=3.524(1) Å, c=4.249(2) Å, $\beta \;=\;93.75{\left(3\right)}^{\circ }$, and volume V=70.8(1) Å${}^{3}$. The variation with pressure of the orthorhombic cell parameters and volume are shown in Figure 3b,c, and those of the monoclinic cell in Figure S13 of the SI. These parameters vary continuously with pressure, confirming that phase A is stable in this pressure range. Figure 3c also compares the volume of phase A to that of the AA-II (*P2/c*) phase reported in Ref. [14]. It may be seen that the volume of the *Ima2* cell is bracketed by four and five times the volume of AA-II. If we assume that phase A is composed of the same formula units as AA-II and four times as much units, the volume reduction at the transition would be about 12%, which seems rather large. This supports the fact that phase A is composed of different moieties than AA. It can also be observed that the compressibility of phase A is similar to that of AA-II, which suggests that phase A is a molecular solid.

### 2.4. Transformation of Pure AA

#### 2.4.1. Raman Experiments

**Figure 4:**
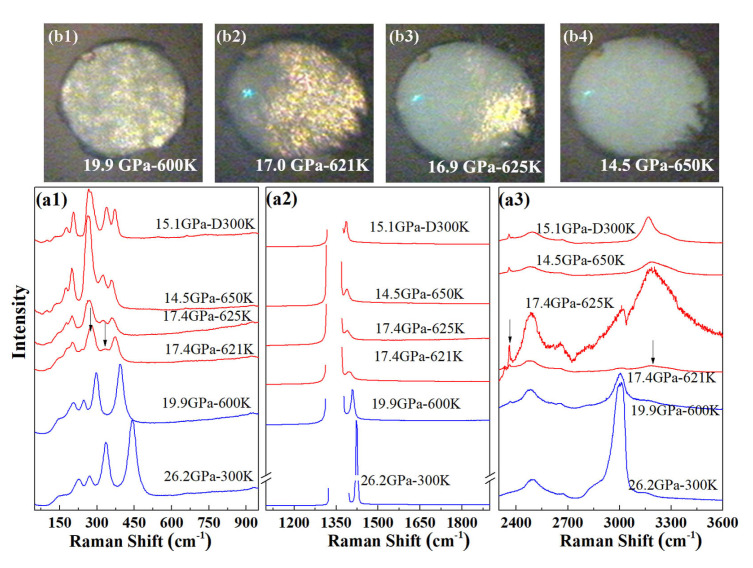
High *P*-*T* transformation of pure AA. (**a**) Raman spectra collected before (blue lines) and during (red lines) the transformation (621–650 K). The uppermost spectrum was collected after cooling back to 300 K. The arrows in the figure indicate the positions of new peaks. “D” indicates that the measurement was performed on decreasing temperature; (**b**) Photographs of the sample chamber before (600 K) and during the transformation (621–650 K).

We carried out high-temperature Raman experiments on pure AA in order to determine whether the formation of phase A requires the participation of nitrogen. Starting from a sample at 26.2 GPa at 300 K, the temperature was increased in several steps. The measured Raman spectra are shown in Figure 4. Upon heating to 600 K, the sample pressure slowly reduced to 20 GPa. As for the AA + N${}_{2}$ system, we found no evidence for a phase transition in AA below 600 K and around 20 GPa. When the temperature was further increased to 620 K, the pressure in the sample chamber reduced to 17.4 GPa and a clear change of the Raman spectrum took place: first, the Raman band peaked at 277 cm${}^{-1}$ split and new Raman bands appeared at 332 cm${}^{-1}$ and 2359 cm${}^{-1}$; second, the N-H stretch bands discontinuously shifted to higher frequencies. After increasing temperature to 650 K, no more change was detected in the Raman spectrum or on cooling down the sample to 300 K. The visual images presented in Figure 4b also show a loss in white light transmission of the sample occurring concomitantly as the changes in Raman spectrum, starting from one side of the sample chamber at 620 K, and completing by 650 K.

Thus, pure AA transforms to a new phase above 620 K which is referred hereafter as phase B. The sharp lattice and internal modes of the Raman spectrum of phase B are in favor of a crystalline compound. Further Raman measurements were performed on decompression at 300 K from 21.4 GPa to 15.1 GPa, and the peak frequencies as a function of pressure are plotted in Figure 2.

The N-H stretch band can be deconvoluted into two peaks located at 3183 cm${}^{-1}$ and 3281 cm${}^{-1}$ at 20.5 GPa–300 K, and whose frequency increases with pressure. A single N-H bending mode is detected at 1639 cm${}^{-1}$. These N-H vibration frequencies largely differ from those of NH${}_{4}^{+}$ in AA, indicating that NH${}_{4}^{+}$ ions disappeared in the high *P*-*T* transformation. They are in the same frequency range as those of NH${}_{3}$, but, unlike for phase A, the number of modes and their frequencies do not match those of pure solid NH${}_{3}$, which suggests that the latter is not formed. There is also no good match with the Raman modes of N${}_{2}$H${}_{4}$. As for AA + N${}_{2}$, we found no evidence for the formation of H${}_{2}$ in the Raman spectra (see Figure S10 of the SI).

The two peaks at 2368 cm${}^{-1}$ and 2392 cm${}^{-1}$ at 20.5 GPa coincide, both in frequency and pressure shift, with the N≡N stretch of the pure N${}_{2}$ solid, strongly indicating that N${}_{2}$ molecules were formed at the transition and separated from phase B. The Raman peak at 1395 cm${}^{-1}$ at 20.5 GPa is close to, and shifts similarly with pressure as the N=N stretching mode of AA and phase A, indicating that phase B also contains species with doubly bonded nitrogen atoms. As in phase A, no Fermi resonance is observed between this mode and the N-H bending of NH${}_{4}^{+}$, thus confirming that NH${}_{4}^{+}$ are no longer present in phase B. Further comparison of phases A and B will be presented in the next section.

#### 2.4.2. XRD Experiments

**Figure 5:**
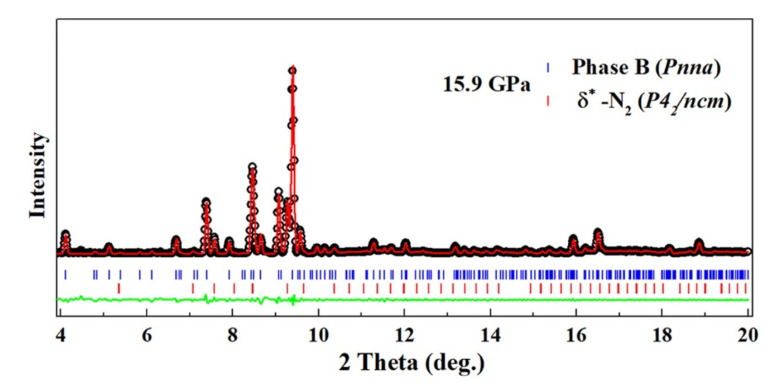
XRD pattern of the pure AA sample at 15.9 GPa–300 K after the high *P*-*T* transformation. The background has been subtracted for easier visualization. Experimental data are shown by red circles. The black line is a two-phase Le Bail fit of the pattern using the *Pnna* unit cell for phase A and $\delta^{*}$-N${}_{2}$ ($P4_{2}/ncm$). The blue line is the fit residual. Ticks indicate the position of the Bragg peaks of each phase.

The XRD pattern of phase B was measured at 24.8 GPa and 15.9 GPa. In both patterns, peaks from solid N${}_{2}$ either in the ϵ or the $\delta^{*}$ phase could be identified, thus confirming the formation of solid N${}_{2}$ in the transformation. The remaining peaks in the pattern at 15.9 GPa were used for unit cell search using DICVOL06 [37]. Here again, two solutions were found compatible with the data; the first one is an orthorhombic unit cell of most probable space group *Pnna* with a=12.799(3) Å, b=8.957(3) Å, c=4.724(1) Å and volume 541.6(3) Å${}^{3}$ at 15.9 GPa; the second one is a monoclinic cell of most probable space group P2/m, a=8.254 Å, b=6.722(1) Å, c=5.356(1) Å, $\beta =91.99{\left(1\right)}^{\circ }$, and volume 296.95(1) Å${}^{3}$. The Le Bail refinement using the *Pnna* cell is shown in Figure 5, and the one for the *P2/m* structure is given in Figure S14 of the SI. The orthorhombic cell better fits the data, and is thus the preferred solution. The volume ratio with respect to AA-II is 10.7, which suggests at least ten times more formula units. However, this large volume could also signal that phase B is not a single phase.

## 3. Discussion

The experimental results that are presented above show that AA ceases to be stable and undergoes a transformation for P>20 GPa and T>620 K, whether it is or not embedded in N${}_{2}$. However, the transformation gives a different product in the presence of N${}_{2}$, judging both from the Raman spectra and XRD patterns. It may be conjectured that phase A is obtained in two steps, first from the transformation of AA into phase B, and second from the reaction of phase B with N${}_{2}$. Below, we first compare the vibrational and structural properties of phases A and B, and then compare the two with predicted stable N${}_{x}$H${}_{y}$ compounds in the literature.

### 3.1. Comparison of Phases A and B

**Figure 6:**
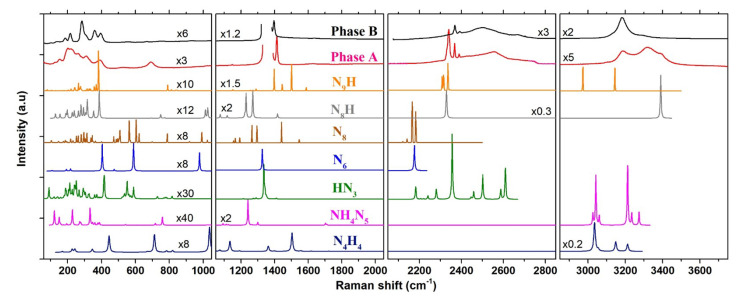
Comparison between the experimental Raman spectra of phases A and B at 20.5 GPa–300 K, and the theoretical ones of N${}_{4}$H${}_{4}$ (*P2${}_{1}$/c*), NH${}_{4}$N${}_{5}$ (*Pbcm*), HN${}_{3}$ (*Cc*), N${}_{6}$ (*C2/m*), N${}_{8}$ (*P1*), N${}_{8}$H (*P-1*) and N${}_{9}$H (*P1*) at 20 GPa–0 K, computed by DFPT. The structures used for the Raman calculations are from Ref. [15] for NH${}_{4}$N${}_{5}$, Ref. [38] for N${}_{6}$, Ref. [39] for N${}_{8}$, Ref. [40] for HN${}_{3}$, Ref. [4] for N${}_{4}$H${}_{4}$ N${}_{8}$H and Ref. [5] for N${}_{9}$H. The frequency range was divided into four panels for easier visualization. The vertical scale of each panel is the same but individual scale factors were applied as indicated to some spectra in order to increase the visibility of peaks.

The comparison of the Raman spectra of phases A and B at 20.5 GPa at 300 K, reveal significant differences, as seen in Figure 2 and Figure 6. First, as noted above, there are clear differences in the number and frequency vs. pressure evolution of the N-H stretch bands. In phase A, this band is composed at least of five peaks, but only 1 of them, located at ∼3110 cm${}^{-1}$ and nearly invariant with pressure was assigned to the new phase, as the 4 others match those of solid NH${}_{3}$. As a matter of fact it is possible that other peaks overlapping with those of solid NH${}_{3}$, are present but not resolved. In phase B, two N-H stretch modes are observed which differ in frequency and pressure shift from those of phase A. In both phases though, we found that these modes are not compatible with the N-H stretch of the NH${}_{4}^{+}$ ion, which signals their disappearance in the transformation and likely conversion into ammonia molecules or other species with N-H radicals.

Another marked difference in the Raman spectra resides in the Raman bands at ∼700 cm${}^{-1}$ and 2320–2340 cm${}^{-1}$ in phase A, which are not present in phase B. The first one is difficult to assign as it may arise from several possible vibrational motions. One the one hand, the frequency of this mode is similar to that of the N-N stretching vibration in the extended covalent nitrogen solid cg-N [1]; however, the Raman spectrum and compressibility of phase A suggests that it is a hydronitrogen molecular solid, so the N-N stretch would rather be expected around 1100–1150 cm${}^{-1}$ as in hydrazine [35]. The latter also presents a Raman mode close to 700 cm${}^{-1}$ at 25 GPa (see Figure 2), which corresponds to a torsion of the N-N bond through rocking of the NH${}_{2}$ units. Examining the vibrational modes that were obtained by our calculations for several N${}_{x}$H${}_{y}$ compounds (see below), we also found that this mode is compatible with other types of motion such as the torsion (in N${}_{3}$H and N${}_{5}$H) or the bending (in N${}_{6}$ and N${}_{8}$) of N-N-N units. The second band at 2320–2340 cm${}^{-1}$, composed of two peaks, is located in the range of frequency of the N≡N stretch of the N${}_{2}$ molecule and thus likely comes from the presence of this molecule inside the crystal structure of phase A. N${}_{2}$ molecules are also formed in the transformation of pure AA, but, in this case, separate from phase B. This suggests that the N${}_{2}$ molecules are not interstitial in phase A, and that they are not formed in sufficient amount in the transformation of pure AA to reach the required stoichiometry of phase A. This would confirm that excess N${}_{2}$ is required to obtain phase A from AA. It is also interesting to note that a shifted N${}_{2}$ Raman vibron was observed in the reaction products of compressed N${}_{2}$-H${}_{2}$ mixtures [8,9,10], and assigned to trapped N${}_{2}$ molecules.

### 3.2. Comparison with Predicted N${}_{x}$H${}_{y}$ Compounds

In order to establish the composition of the two newly formed phases A and B resulting from the transformation of AA at high *P*–*T*, we compared their Raman spectra and XRD patterns to those of poly-hydronitrogen N${}_{x}$H${}_{y}$ compounds predicted as stable in the literature. We limited the comparison to compounds which have been predicted as stable below 60 GPa. Furthermore, we only present below results for compounds with a nitrogen/hydrogen content ratio equal or superior to AA. As a matter of fact, to the best of our knowledge, only two H-rich compounds besides ammonia have been reported so far to be stable in calculations below 60 GPa, of respective formula NH${}_{4}$ and NH${}_{5}$ [4]. Both contain H${}_{2}$ molecules and, as shown in Figure S10, have strong associated Raman peaks that are not observed in our experiments. The XRD the patterns were obtained from the optimized structure at 20 GPa, and the Raman spectra were computed using density-functional perturbation theory, as described in the Methods section. Figure 6 shows the comparison for the Raman spectra, and Figure S15 of the SI compares the XRD patterns.

*N${}_{4}$H${}_{4}$*. A compound of same stoichiometry as AA, but composed of tetrazene (NH${}_{2}$-N-N-NH${}_{2}$) molecules was predicted by computational studies to be stable above 36 GPa [4,11]. Our previous study showed that this compound is less stable than AA-II in calculations below 102 GPa, and it is not observed in experiments up to 85 GPa at 300 K [14]; however, the transition may be facilitated at high temperature. The present calculations were made using the*P2${}_{1}$/c* structure that is reported in Ref. [4]. The comparison of the computed Raman spectra with phases A and B show strong differences, especially in the 1000–1600 cm${}^{-1}$ region, and the same is true for the XRD pattern; we may thus safely rule out the formation of this compound in our experiments.

*NH${}_{4}$N${}_{5}$*. As stated in the introduction, a motivation of this work was the theoretical prediction [15] that ammonium azide will react with N${}_{2}$ and transform into ammonium pentazolate (NH${}_{4}$N${}_{5}$) above 12.5 GPa. The recent synthesis of the metal pentazolate salts CsN${}_{5}$ [16] and LiN${}_{5}$ [17] from the direct reaction of, respectively, CsN${}_{3}$ and Li with N${}_{2}$ at high *P*-*T* gave credit to this prediction. According to Ref. [15], the stable phase of NH${}_{4}$N${}_{5}$ at this pressure is of Pbcm symmetry and it is composed of hydrogen-bonded NH${}_{4}^{+}$ and cyclo-N${}_{5}^{-}$ ions. In the DFPT calculated Raman spectrum of this structure at 20 GPa, the distinctive features of th N${}_{5}^{-}$ ion are the breathing (1240 cm${}^{-1}$), bending (758 cm${}^{-1}$), and deformation (1090–1125 cm${}^{-1}$) vibrations. The frequencies of these modes in NH${}_{4}$N${}_{5}$ obtained from our calculations compare very well with those measured in LiN${}_{5}$ [17] and CsN${}_{5}$ [16]. There is no corresponding mode in the Raman spectra of phases A and B, apart maybe for the peak of phase A at 692 cm${}^{-1}$, but the predicted strong breathing mode at 1240 cm${}^{-1}$ is definitely not observed, as seen in Figure 6. Thus, we may conclude that NH${}_{4}$N${}_{5}$ has not been formed in our experiments and that the synthesis of this compound at high *P*–*T* is not as straightforward as for the metal pentazolate salt. The reaction might require a higher pressure or temperature to overcome the kinetic barrier, however the decomposition of AA in liquid N${}_{2}$ observed in this work limits the temperature range of investigation to below the melting line of N${}_{2}$.

*HN${}_{3}$*. The disappearance of NH${}_{4}^{+}$ ions in the high *P*–*T* transformation of AA + N${}_{2}$ and the observation of NH${}_{3}$ as a reaction product could indicate that proton transfers from NH${}_{4}^{+}$ to N${}_{3}^{-}$ occur during the transformation to yield HN${}_{3}$. To the best of our knowledge, the crystal structure of the HN${}_{3}$ solid has not been investigated at high pressure, but theoretical studies [4,5] have predicted a *P2${}_{1}$/c* structure composed of long chains with N-N bond lengths in the range of single and double bonds. The XRD pattern and Raman spectrum of this structure reported in Ref. [5] are very different from that observed, so we did not consider it. Instead, we assumed that the crystal structure remains the same as that determined at ambient pressure [40]. The latter, of Cc symmetry, consists of 16 HN${}_{3}$ molecules per unit cell connected to each other by N-H⋯N hydrogen bonds. We note that this structure is dynamically stable at 20 GPa in our calculations, since all of the vibrational modes have positive frequencies. As seen in Figure 6, the calculated Raman spectrum of the *Cc* HN${}_{3}$ solid also largely differ from those of phases A and B; in particular, the N-H stretching modes are located at much lower frequencies (2350–2610 cm${}^{-1}$) and the strongest N=N stretch peak (1336 cm${}^{-1}$) is also 70 cm${}^{-1}$ below. Although there remains uncertainty regarding the correct structure of HN${}_{3}$ at 20 GPa, we thus consider that it is not a good candidate for the reaction products of AA + N${}_{2}$.

*N${}_{6}$ and N${}_{8}$*. Recent theoretical studies have predicted the existence of two new allotropes of nitrogen in the form of N${}_{6}$ [38] and N${}_{8}$ [39] molecules. Both molecules exhibit several resonnance structures mixing single, double, and triple nitrogen bonds. The predicted crystal structures for N${}_{6}$ (*C2/m*) and N${}_{8}$ (*P1*) were found more stable than the cg-N solid below 20 GPa. The experimental synthesis of the N${}_{8}$ solid was recently claimed by compressing hydrazinium azide [(N${}_{2}$H${}_{5})($N${}_{3}$)] above 40 GPa, decomposing to N${}_{2}$ below 20 GPa [41]. The theoretical Raman spectrum of the N${}_{8}$ crystal, computed here at 20 GPa while using the structure reported in Hirshberg et al. [39], contains many (96) active modes due to the low symmetry of this structure. The Raman spectra of phases A and B are much simpler than that of N${}_{8}$ and many predicted peaks for this solid are not observed. Moreover, the predicted frequency range for the N≡N stretch modes (2120–2180 cm${}^{-1}$) in N${}_{8}$ is lower than that observed in both phases A and B. The N${}_{6}$ crystal has a higher symmetry (*C2/m*) and thus a simpler Raman spectrum, yet the Raman peaks occur at similar frequencies as in N${}_{8}$ and, thus, differ from those of phases A and B. Thus, we may safely conclude that these species are not formed in the transformation of AA. We also note that the calculated Raman spectrum of N${}_{8}$ largely differ from that measured in Duwal et al. [41] (in particular, for the N≡N stretch observed around 2380 cm${}^{-1}$), casting doubts that these authors actually produced N${}_{8}$ by compression of (N${}_{2}$H${}_{5}$)(N${}_{3}$).

*N${}_{8}$H and N${}_{9}$H*. N${}_{8}$H (*P-1*) and N${}_{9}$H (*P1*) are two hydronitrogen compounds that emerged from theoretical structural searches, in Refs. [4,5], respectively. These two compounds have in common the presence of N${}_{2}$ molecules in their crystal structure, which we suspect to be the case in phase A. N${}_{8}$H is also composed of N${}_{5}$H moieties, whereas N${}_{9}$H contains infinite zig-zag chains of N and H atoms. The computed Raman spectra for these two solids at 20 GPa display intense peaks around 2330 cm${}^{-1}$ coming from N${}_{2}$ molecules. Interstingly, there is a good match with the spectrum of phase A, supporting our intuition that N${}_{2}$ molecules are part of the crystal structure of phase A. N${}_{9}$H also presents Raman peaks which matches well with the N=N and N-H stretch of phases A and B; however, some strong predicted peaks, such as those at 1500 cm${}^{-1}$ and 2973 cm${}^{-1}$, are not observed, and the XRD pattern of the *P1* N${}_{9}$H solid does not match those measured for phases A and B (Figure S15).

## 4. Conclusions

In conclusion, the present experimental investigation of ammonium azide at high pressure and temperature showed, first, that this compound decomposes in liquid N${}_{2}$ at 13 GPa–700 K, into N${}_{2}$ and NH${}_{3}$; second, that it transforms to new phases for P>20 GPa and T> 620–650 K. This transformation was observed both in pure AA and in AA embedded in N${}_{2}$, but the final products are different in the two cases. In pure AA, a new crystalline phase, called phase B, is formed mixed with solid N${}_{2}$. For AA + N${}_{2}$, we determined that the products are more likely composed of a new crystalline phase, called phase A, which is mixed with solid ammonia and the excess N${}_{2}$ solid. Phases A and B could both be recovered at ambient temperature and were found stable to at least 7 GPa (A) and 15 GPa (B). The XRD patterns of these solids have been collected on varying pressure at 300 K, showing well defined crystalline peaks in the two cases. Candidate structures have been disclosed with larger unit cell volumes as compared to the AA solid. To establish the content of these solids, the Raman spectrum was measured over the 7–25 GPa range. A common feature of the two phases appears to be the loss of ammonium ions and the presence of doubly-bonded nitrogen species. In addition, phase A presents Raman bands assigned to N≡N stretch and N=N bend which are not observed in phase B. The first one are more likely the signature of N${}_{2}$ molecules in the crystal structure. The Raman spectra and XRD patterns were finally compared to nitrogen and hydronitrogen compounds predicted in the literature as stable at high pressure. No correspondence was found for any of the considered compounds; however, similar vibrational features were found with the N${}_{9}$H solid, composed of N${}_{2}$ molecules and infinite zig-zag chains. This study thus shows that new hydronitrogen compounds may be discovered in a moderate *P*-*T* range. Future work will aim to establish the nature and structure of the newly produced crystalline solids.

## Supplementary Materials

The following are available online at https://www.mdpi.com/1996-1944/13/18/4102/s1, Figure S1: Raman spectrum of the AA sample embedded in N${}_{2}$ at 6 GPa–300 K after loading in the DAC; Figure S2: Experimental paths mapped in the nitrogen phase diagram; Figure S3: Photographs of the sample chamber and Raman spectrum of AA + N${}_{2}$ along path 1; Figure S4: Raman spectrum at different positions in the sample chamber at 12.6 GPa–300 K after the decomposition of AA in liquid N${}_{2}$ at 700 K; Figure S5: Raman spectrum of AA + N${}_{2}$ collected along path 2; Figure S6: Raman spectrum of phase A upon heating to 650 K around 20 GPa; Figure S7: Comparison of Raman spectra in paths 2 and 3; Figure S8: Raman spectrum of phase A at different positions in the sample chamber; Figure S9: Raman spectrum of phase A upon decompression at 300 K; Figure S10: Extended Raman spectra of phase B and comparison with NH${}_{4}$ and NH${}_{5}$; Figure S11: XRD pattern (a) and image (b) of phase A (a); Figure S12: Measured cell parameters and volume of N${}_{2}$ and NH${}_{3}$ solids mixed with phase A as a function of pressure at 300 K; Figure S13: XRD pattern of the AA + N${}_{2}$ sample at 20.1 GPa–300 K after the high *P*-*T* transformation; Figure S14: XRD pattern of the pure AA sample at 15.9 GPa–300 K after the high *P*-*T* transformation; Figure S15: Comparison between the experimental XRD patterns of phases A at 20.1 GPa–300 K and Phase B at 15.9 GPa–300 K, with the theoretical ones of N${}_{4}$H${}_{4}$, N${}_{6}$, N${}_{8}$, N${}_{8}$H, N${}_{9}$H, HN${}_{3}$ and NH${}_{4}$N${}_{5}$ at 20 GPa–0 K.

## Abbreviations

The following abbreviations are used in this manuscript:

*P*	Pressure
*T*	Temperature
HEDM	High energy-density material
AA	Ammonium azide
FWHM	Full width at half maximum
DFT	Density functional theory
DFPT	Density functional perturbation theory
