# Supplementary material for: Transformation of Ammonium Azide at High Pressure and Temperature
Source: Materials (Basel). 2020 Sep 15;13(18):4102. doi: 10.3390/ma13184102 (PMC7560398; doi:10.3390/ma13184102)
Supplement: Supplementary file 1 [file materials-13-04102-s001.pdf]

materials

Supplementary Materials: Transformation of Ammonium Azide at High Pressure and Temperature

Guozhao Zhang, Haiwa Zhang, Sandra Ninet, Hongyang Zhu, Keevin Beneut, Cailong Liu, M. Mezouar, Chunxiao Gao and Frédéric Datchi

1. Sample Characterization after DAC Loading in N_2.

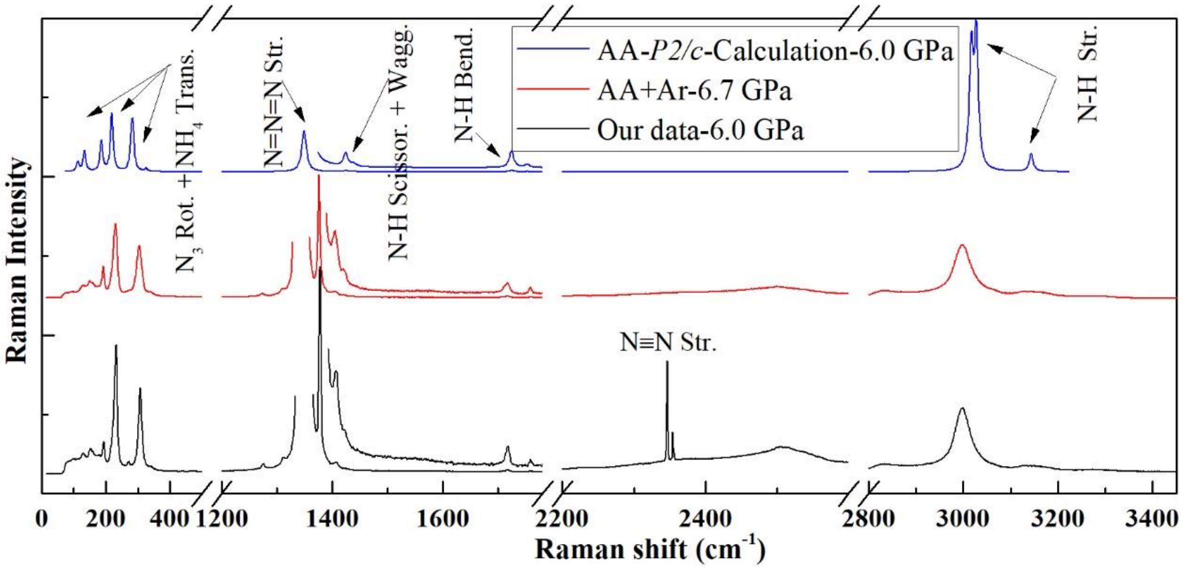

Figure S1. The Raman spectrum of the AA sample embedded in N_2 at 6 GPa—300 K after loading in the DAC is compared to our previous experimental data of AA in argon and theoretical calculations as reported in Ref.1 [1]. The stable phase of AA at this P-T condition is AA-II of structure P2/c.

2. Raman Experiments (AA+N_2)

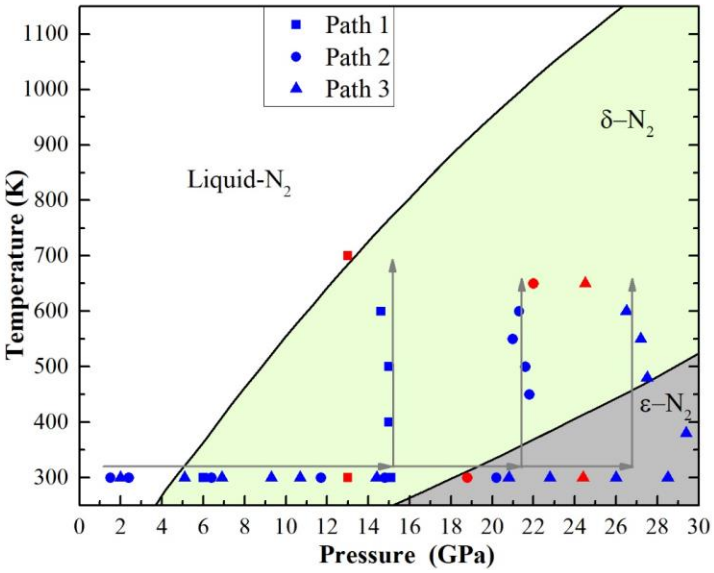

Figure S2. Experimental paths are mapped in the nitrogen phase diagram. Gray arrows represent the experimental paths followed in present Raman experiments; Square, circle, and triangle symbols represent data points for paths 1,2 and 3, respectively. The symbol color indicates the state of the sample: blue is for untransformed AA, red is for the transformed sample.

2.1. Path1 (Temperature Increase at 15 GPa)

Along path 1, the starting pressure at 300 K was 15 GPa and we increased temperature to 700 K at a rate of 100 K per step. The photographs and Raman spectra are shown in Figure S3. As the temperature increases, the pressure in the sample chamber slightly decreased. Until 600 K, the morphology and Raman spectrum of the sample didn't significantly change, showing that AA and N_2 in the sample chamber are stable and no reaction has occurred. When the temperature was increased to 700 K, the pressure in the sample chamber decreased to 13.0 GPa. At these P–T conditions, nitrogen melted, and AA decomposed rapidly. Only two Raman bands were then observed in the Raman spectrum, peaked at 2354 cm^{-1} and 2363 cm^{-1} at 13 GPa and 700 K [2,3], which correspond to the nitrogen triple bond stretching of molecular N_2.

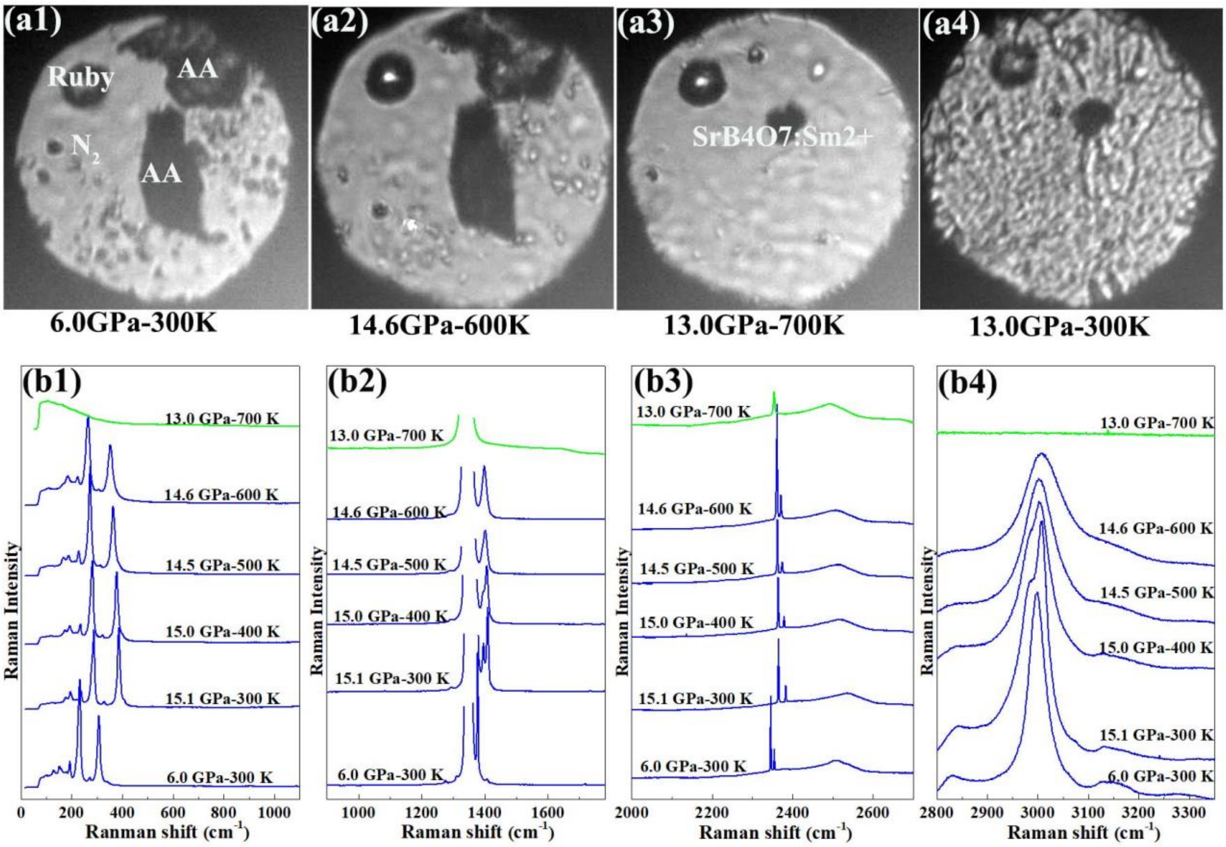

Figure S3. Photographs of the sample chamber (**a**) and Raman spectra of AA+N_2 along path 1.

Raman measurements were taken at different locations in the sample chamber after the temperature reverted to room temperature, and the pressure was 12.6 GPa (Figure S4). In addition to the N≡N stretching band from nitrogen found at all positions, Raman bands were observed in the 3200–3500 cm^{-1} regions at several positions (2, 6 and 8 in Figure S4). These Raman frequencies are similar to those caused by N-H stretching vibration in ammonia [4]. The reason why NH_3 was not detected at high temperature likely comes from its dilution in the N_2 liquid, the small AA sample volume which was loaded compared to N_2, and weaker Raman intensity at high T. At room T, N_2 and NH_3 phase separate in the solid phase (hence the inhomogeneous aspect of the sample at 13 GPa-300 K shown in panel (a4) of Figure S3), and a stronger Raman signal from the NH_3 solid is recorded in sample regions where NH_3 crystallized. We thus conclude that the AA sample dissolved in liquid nitrogen and decomposed into nitrogen and, most likely, ammonia.

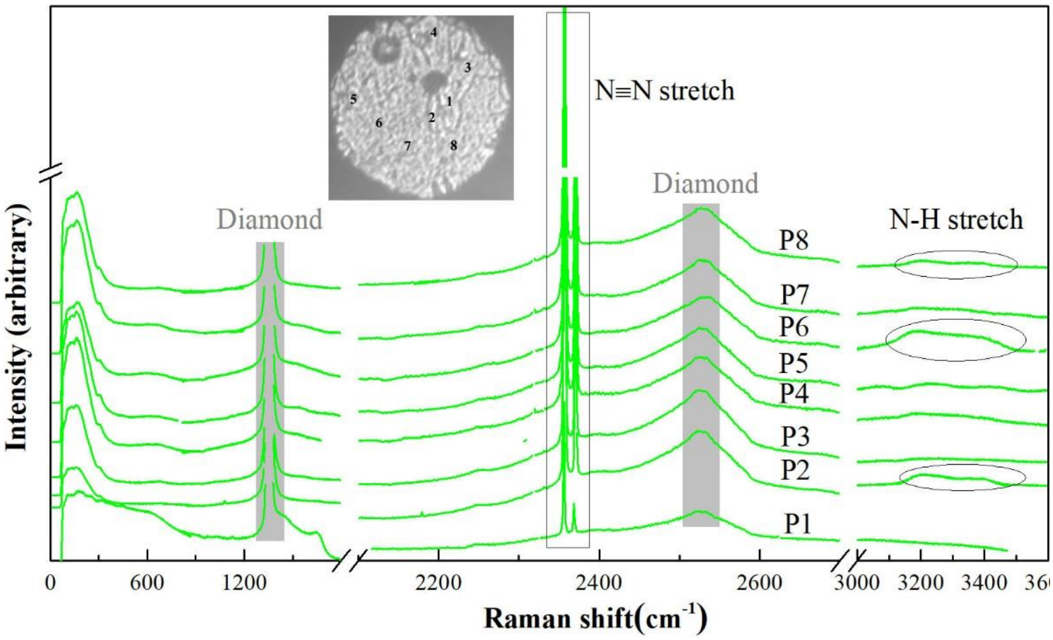

Figure S4. Raman spectrum at different positions in the sample chamber at 12.6 GPa—300 K after the decomposition of AA in liquid N_2 at 700 K. Px stands for different positions in the sample chamber corresponding to the number x in the illustration.

2.2. Path 2 (Temperature Increase at 20 GPa)

In Path 2 experiment, the starting pressure was 20 GPa and we increased temperature to 650K at a rate of 50 K per step. The collected Raman spectra are shown in Figure S5. Until 600 K, the AA and N_2 in the sample chamber was stable. When the temperature was increased to 650 K, the sample Raman signal became very weak. We kept the temperature at 650 K for 10 minutes and then cooled to room temperature. It turned out that the rapid weakening of the sample signal was caused by the failure of the Raman measurement system. After repair, we collected the Raman spectrum of the sample at 18.8 GPa—300 K, which revealed the transformation of the AA sample as discussed in the manuscript. We then reheated the sample to 650 K. As seen in Figure S6, the Raman spectrum of the sample remained basically unchanged during heating, except for some variations in peak intensities.

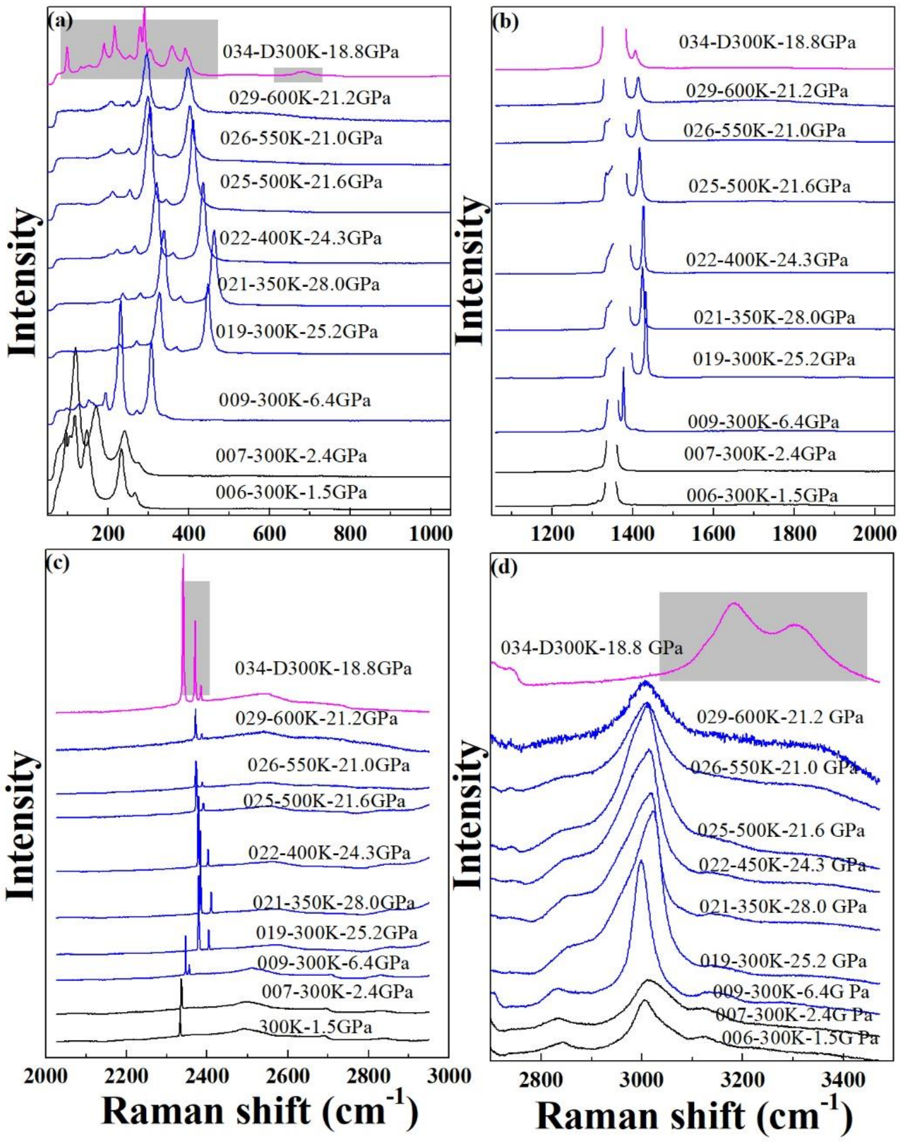

Figure S5. Raman spectra of AA+N_2 collected along path 2; the gray colored regions represent frequency windows where the Raman spectrum of the sample changes.

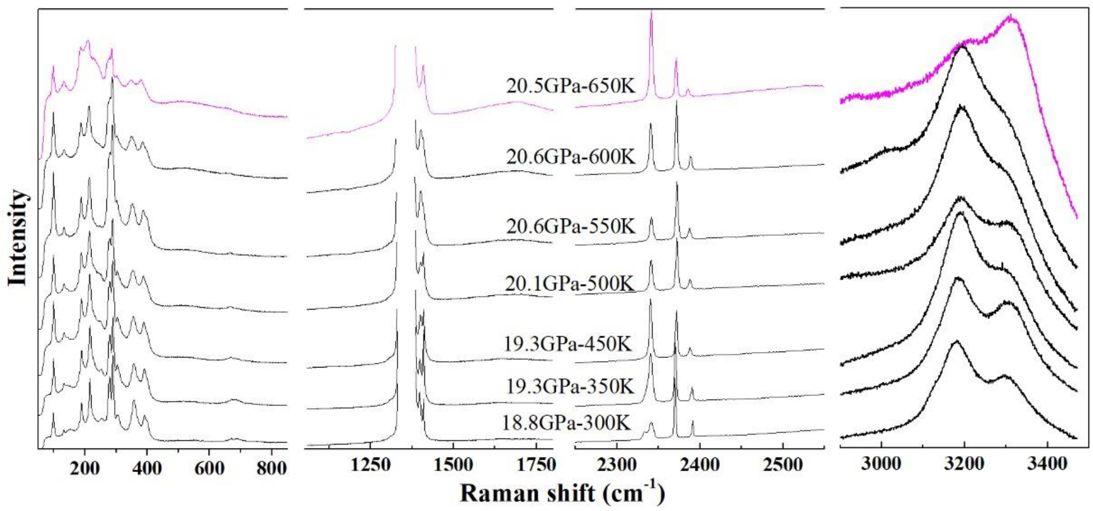

Figure S6. Raman spectrum of phase A upon heating to 650 K around 20 GPa.

2.3. Comparison between Experimental Results of Path 2 and 3

Figure S7 compares the measured Raman spectra of paths 2 and 3 at 650 K, showing that the same transformation was observed at 20.5 GPa and 24.5 GPa.

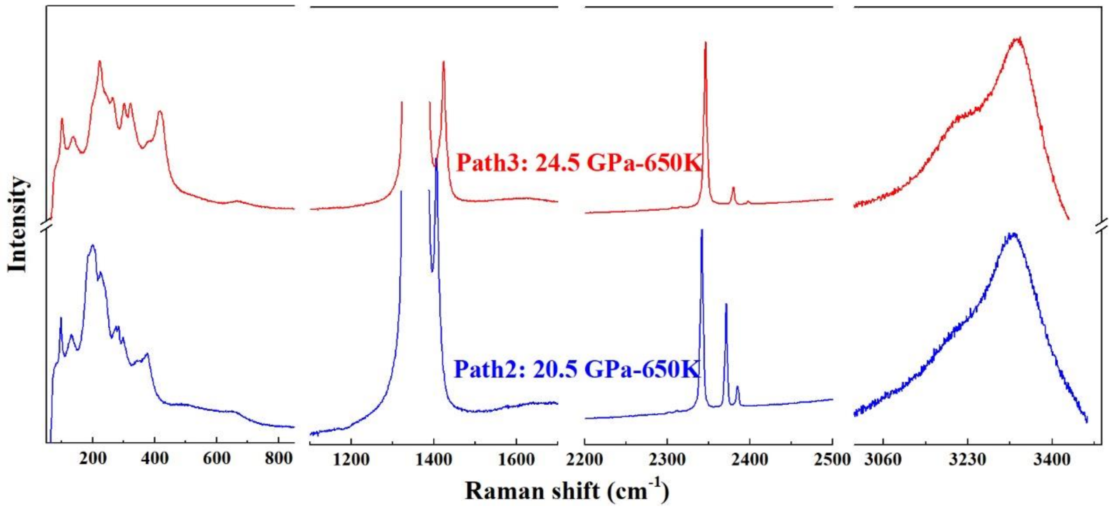

Figure S7. Comparison of Raman spectra in paths 2 and 3.

2.4. Homogeneity of Phase A Sample Abtained in Path 3

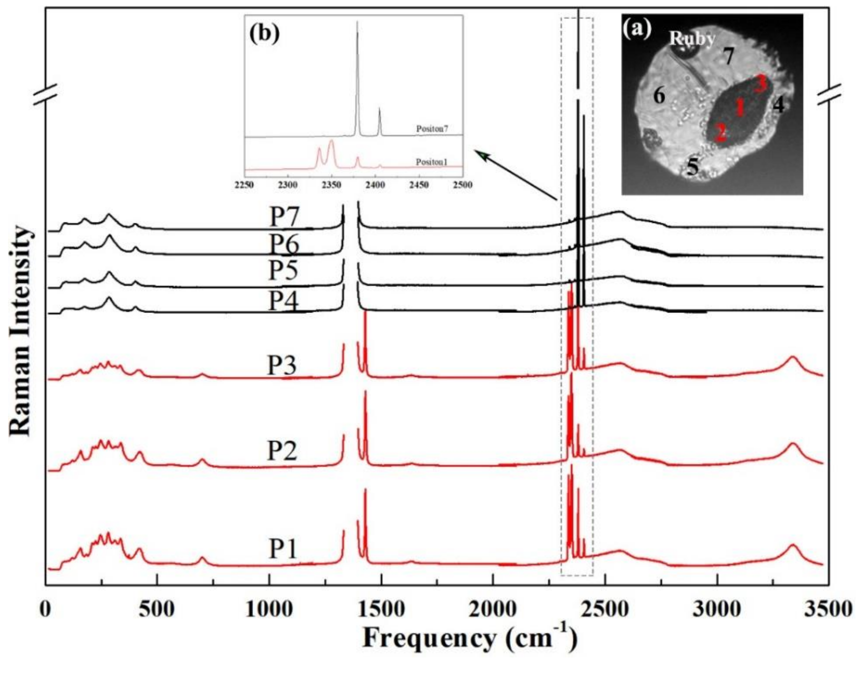

Figure S8. Raman spectra of phase A at different positions in the sample chamber, Px represents different positions in the sample chamber corresponding to the number x in the illustration (**a**); Illustration(**b**) is an enlargement of the Raman peak in the dotted rectangle.

2.5. Raman Spectra upon Decompression of Phase A at 300 K (Path 3)

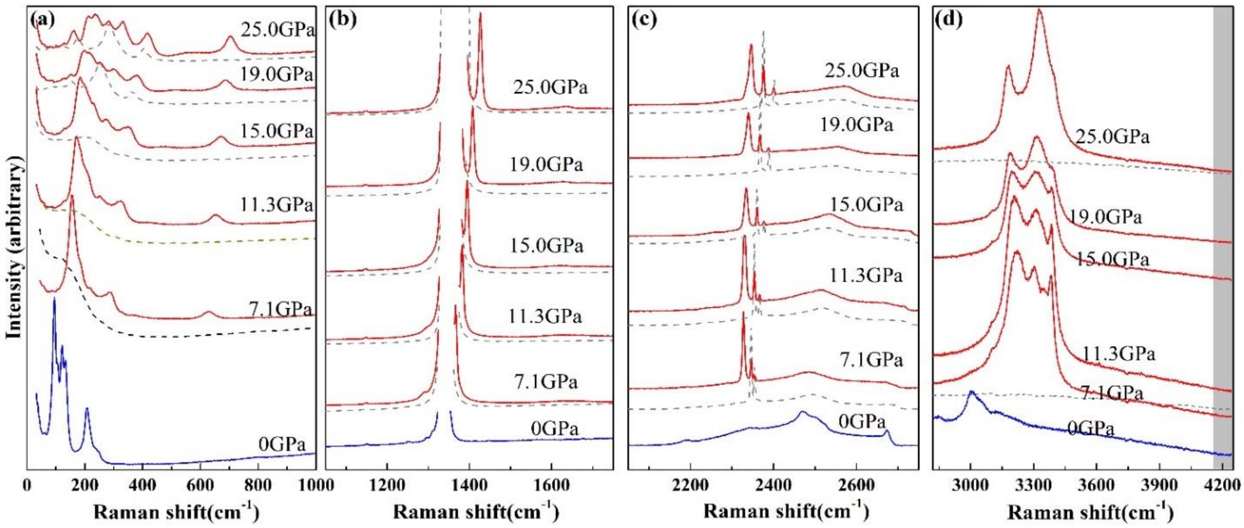

Figure S9. Raman spectra of phase A upon decompression at 300 K. The dashed lines represent the Raman spectra measured from the pure nitrogen solid in the sample chamber. The gray area indicates the frequency range of the Raman band of pure hydrogen (4160–4240 cm^{-1}) in the pressure range of 0–14 GPa.

2.6. Extended Raman Spectra of Phase B and Comparison with NH_4 and NH_5

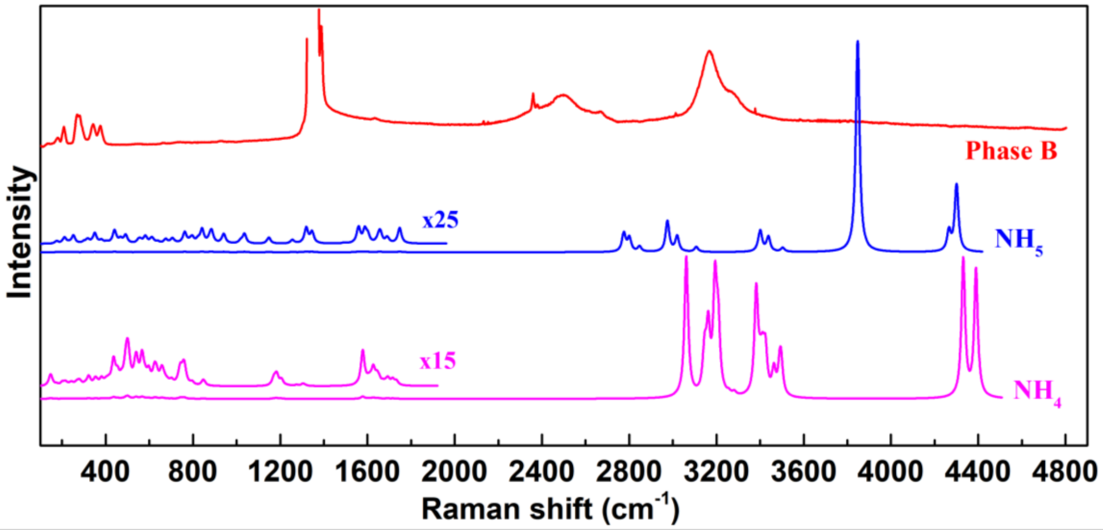

Figure S10. Comparison between the experimental Raman spectra of phase B at 20.5 GPa–300 K, with the theoretical ones of NH_4 (Pc)[9] and NH_5 (C2/c)[9] at 20 GPa–0 K, computed by DFPT.

3. XRD Experiments

3.1. Phase A

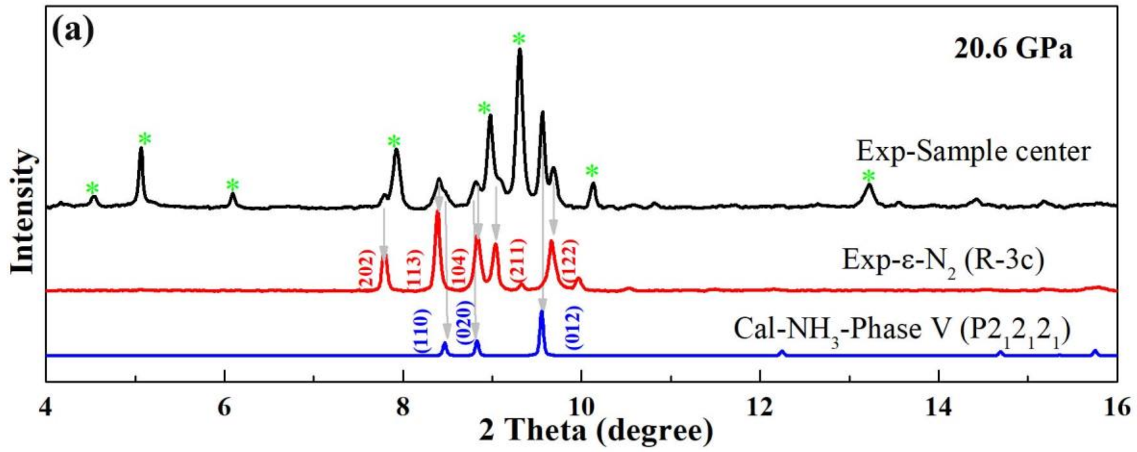

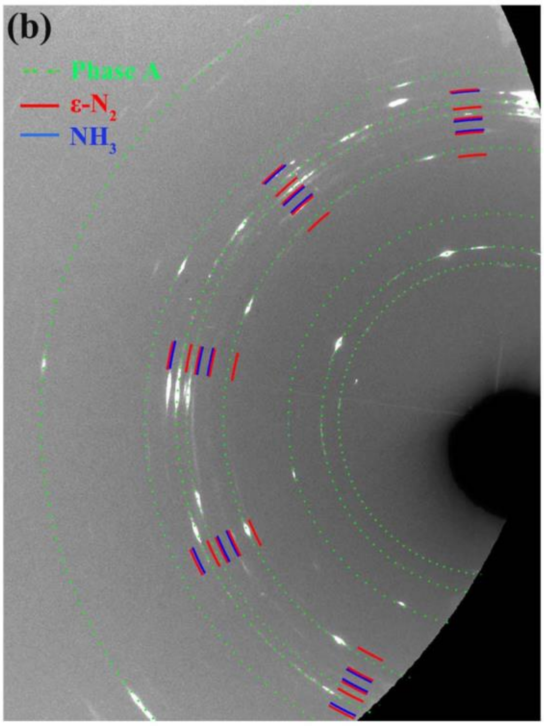

(c)

hkl	2θ(°)	Structure
	4.5364	Phase A
	5.0534	Phase A
	6.7760	Phase A
202	7.7744	N_2
	7.9071	Phase A
113	8.3935	N_2
110	8.4555	NH_3
020	8.7938	NH_3
104	8.8380	N_2
	8.9540	Phase A
211	8.9779	N_2
	9.2822	Phase A
012	9.5377	NH_3
122	9.6569	N_2
	13.1870	Phase A

Figure S11. XRD pattern (**a**) and image (**b**) of phase A at 20.6 GPa – 300 K. In (a) the pattern is compared to those of solid ammonia V (simulated) and ε-nitrogen (measured next to the sample). Green asterisks mark the XRD peaks of phase A; In (b), the dotted green, dashed red and blue circles mark the diffraction rings of phase A, nitrogen and ammonia, respectively. Table (**c**) lists the peak positions of phase A, N_2 and NH_3.

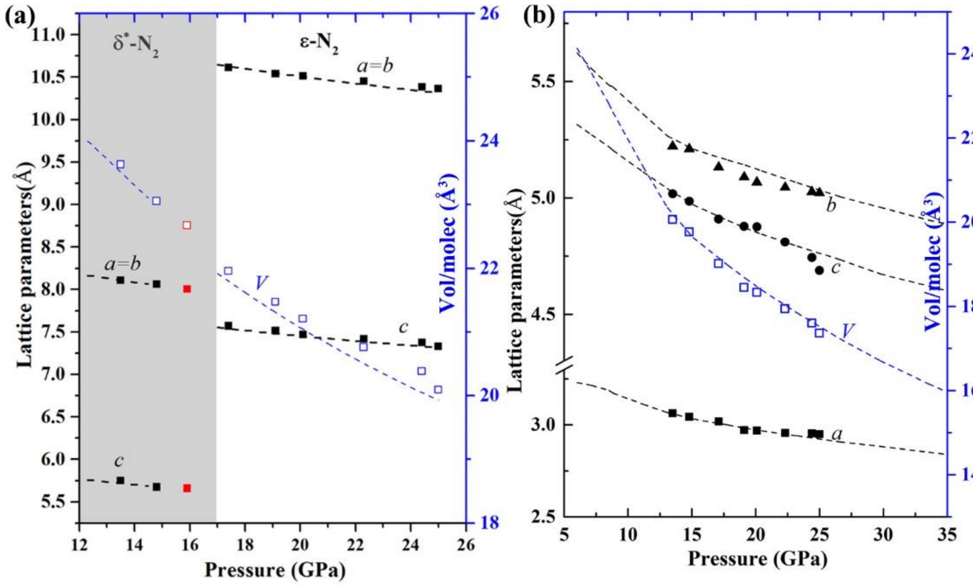

Figure S12. Measured cell parameters and volume of (**a**) N_2 and (**b**) NH_3 solids mixed with phase A as a function of pressure at 300 K. The dotted lines represent the data from literature (Ref. [6] for ε-N_2, Ref. [7,8] for δ*-N_2 and Ref. [5] for NH_3.), and the symbols represent our experimental data. The red-colored symbols come from the Le Bail fitted refinement shown in Figure S5b.

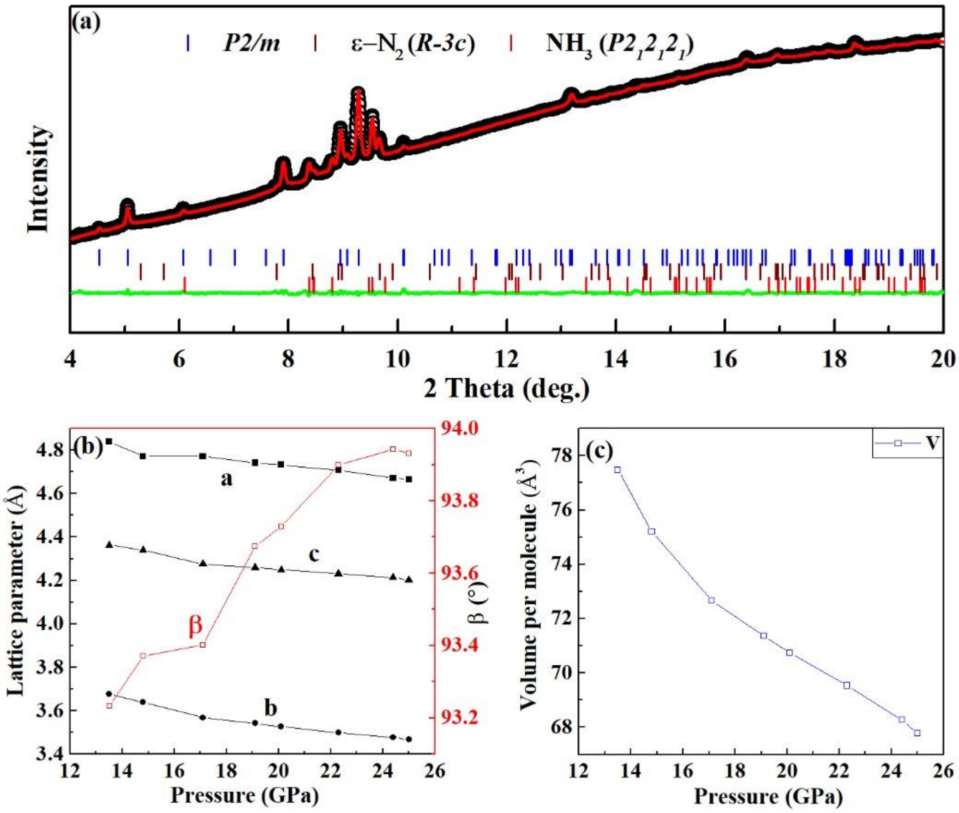

Figure S13. XRD pattern of the AA+N_2 sample at 20.1 GPa–300 K after the high P-T transformation. Panel (**a**) shows the three-phases Le Bail fit of the pattern using the P2/m unit cell for phase A, ε-N_2 (R-3c) and NH_3-V ($P2_12_12_1$). The black circles are experimental data, the red line is the Le Bail fit and the green line is the fit residual. Ticks indicate the position of the Bragg peaks for each phase. The bottom panels show the unit cell parameters (**b**) and volume (**c**) of the P2/m cell.

3.2. Phase B

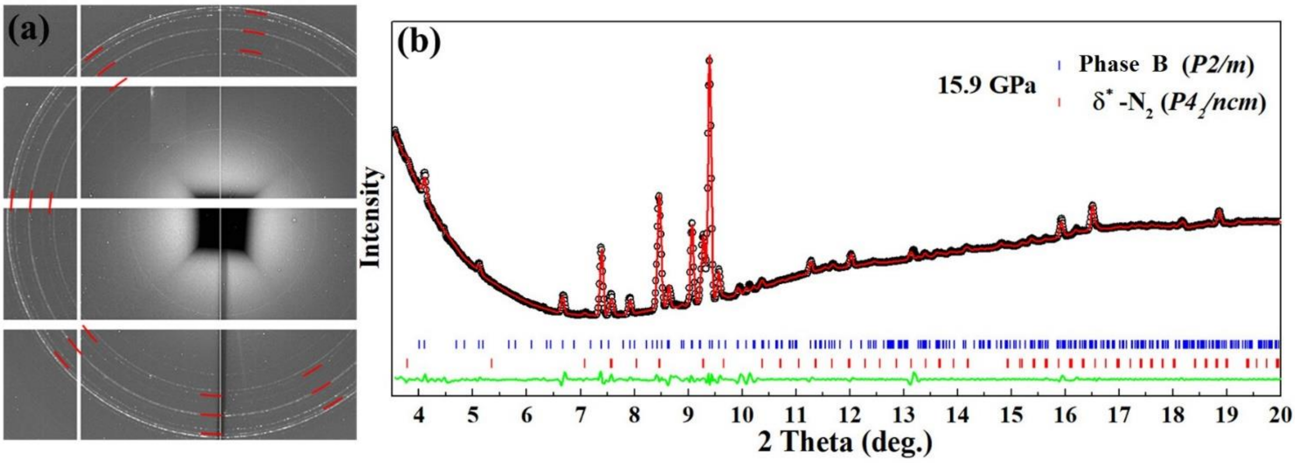

Figure S14. XRD image (**a**) and integrated pattern (**b**) of the pure AA sample at 15.9 GPa-300 K after the high P-T transformation. Experimental data are shown by black circles. The red circles in (a) mark the diffraction rings from δ*-N_2 The red line is a two-phases Le Bail fit of the pattern using the P2/m unit cell for phase A and δ*-N_2 ($P4_2/ncm$). The green line is the fit residual. Ticks indicate the position of the Bragg peaks for each phase.

3.3. Comparison of XRD Patterns of Phases A and B with N_xH_y Compounds

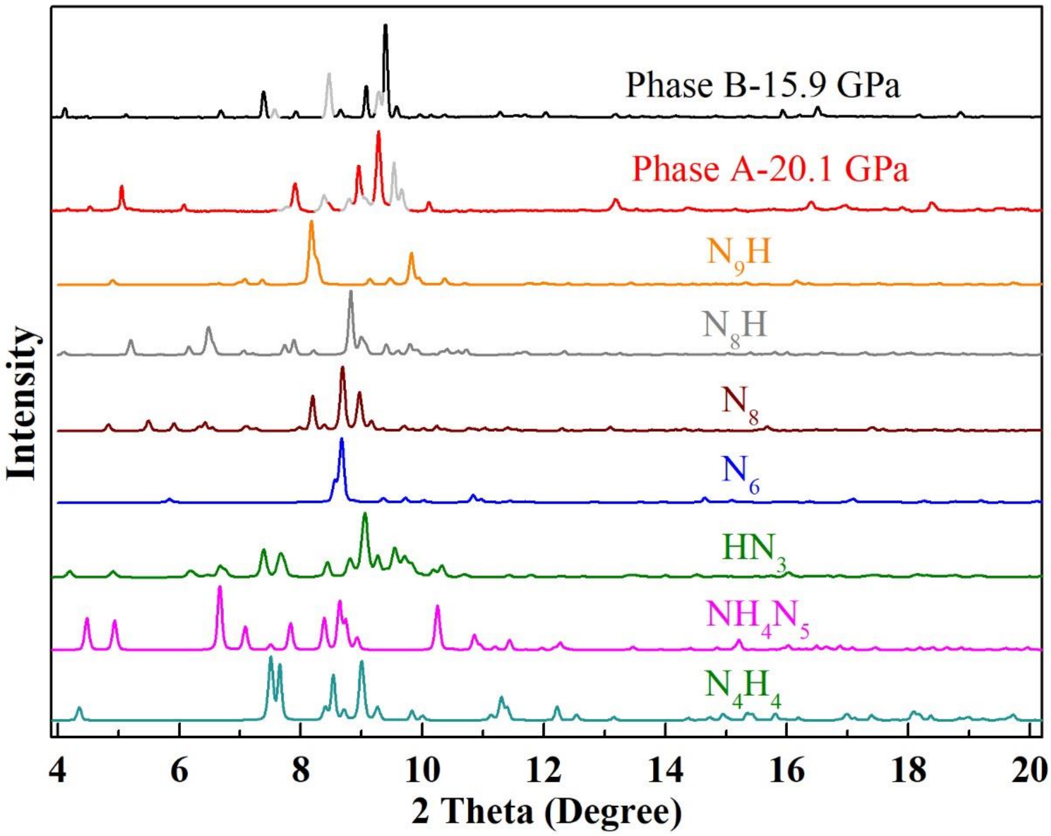

Figure S15. Comparison between the experimental XRD patterns of phases A at 20.1 GPa-300 K and Phase B at 15.9 GPa-300K, with the theoretical ones of N_4H_4 ($P2_1/c$), N_6 (C2/m), N_8 (P1), N_8H (P-1), N_9H (P1), HN_3 (Cc) and NH_4N_5 (Pbcm) at 20 GPa-0 K, computed by DFT (see Figure 6 of the manuscript for the references). The gray colored parts in the XRD patterns of phase A and B indicate peaks from nitrogen and ammonia solids.

References

1. Zhang, G.; Zhang, H.; Ninet, S.; Zhu, H.; Liu, C.; Itié, J.P.; Gao, C.; Datchi, F. Crystal Structure and Stability of Ammonium Azide Under High Pressure. *J. Phys. Chem. C* **2020**, *124*, 135–142, doi:10.1021/acs.jpcc.9b09635.
2. Olijnyk, H.; Jephcoat, A.P. Vibrational Dynamics of Isotopically Dilute Nitrogen to 104 GPa. *Phys. Rev. Lett.* **1999**, *83*, 332, doi:10.1103/physrevlett.83.332.
3. Pu, M.; Liu, S.; Lei, L.; Zhang, F.; Feng, L.; Qi, L.; Zhang, L., Raman study of pressure-induced dissociative transitions in nitrogen. Sol. State Commun. **2019**, *298*, 113645.
4. Ninet, S.; Datchi, F.; Saitta, A. M.; Lazzeri, M.; Canny, B., Raman spectrum of ammonia IV. *Phys. Rev*. B **2006**, *74*, 104101.
5. Datchi, F.; Ninet, S.; Gauthier, M.; Saitta, A.M.; Canny, B.; Decremps, F. Solid ammonia at high pressure: A single-crystal X-ray diffraction study to123GPa. *Phys. Rev. B* **2006**, *73*, 174111, doi:10.1103/physrevb.73.174111.
6. Olijnyk, H., High Pressure X-Ray Diffraction Studies on Solid N2 up to 43.9 GPa. *J. Chem. Phys*. **1990**, *93*, 8968-8972.
7. Hanfland, M.; Lorenzen, M.; Wassilewreul, C.; Zontone, F., Structures of Molecular Nitrogen at High Pressures. *The Review of High Pressure Science Technology* **1998**, *7*, 787-789.
8. Stinton, G. W.; Loa, I.; Lundegaard, L. F.; McMahon, M. I., The crystal structures of δ and δ∗ nitrogen. *J. Chem. Phys*. **2009**, *131*, 104511.
9. Qian, G.-R.; Niu, H.; Hu, C.-H.; Oganov, A. R.; Zeng, Q.; Zhou, H.-Y., Diverse Chemistry of Stable Hydronitrogens, and Implications for Planetary and Materials Sciences. *Sci. Rep*. **2016**, *6*, 25947.
10. Sharma, S. K.; Mao, H. K.; Bell, P. M., Raman Measurements of Hydrogen in the Pressure Range 0.2-630 kbar at Room Temperature. Phys. Rev. Lett. 1980, 44, 886.

© 2020 by the authors. Submitted for possible open access publication under the terms and conditions of the Creative Commons Attribution (CC BY) license (http://creativecommons.org/licenses/by/4.0/).
